# Artificial intelligence, ethics, and hospital medicine: Addressing challenges to ethical norms and patient‐centered care

**DOI:** 10.1002/jhm.13364

**Published:** 2024-04-22

**Authors:** Micah Prochaska, David Alfandre

**Affiliations:** ^1^ Section of Hospital Medicine, Department of Medicine University of Chicago Chicago Illinois USA; ^2^ MacLean Center for Clinical and Medical Ethics University of Chicago Chicago Illinois USA; ^3^ US Department of Veterans Affairs VA National Center for Ethics in Health Care Washington District of Columbia USA; ^4^ Department of Population Health NYU Grossman School of Medicine New York New York USA

## Abstract

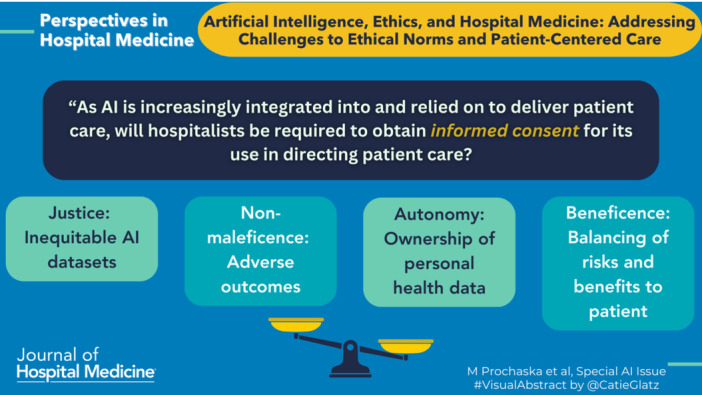

## INTRODUCTION

Recent advances in artificial intelligence (AI) will transform how we care for hospitalized patients.^1^, ^2^, ^3^ Although AI has been present in healthcare for many years,^4^, ^5^ advances in the underlying models and improved computational power will enable AI to be seamlessly integrated into the routine practice of hospital medicine.^4^, ^6^, ^7^ Hospitalists are likely to use AI in a number of ways, including triaging patients, automating progress notes, diagnosing patients, assessing risk, and identifying treatment plans.^8^, ^9^ Since AI models are trained on vast amounts of patient data, they will be able to complete these tasks with increasing speed, accuracy, and reliability—compared to humans or current systems.^2^, ^10^ As with any new disruptive technology with the potential to affect patients' care and their health outcomes, hospitalists should be attentive to the ethical concerns AI may create.

As front‐line providers, hospitalists' attentiveness to ethical concerns will help ensure that the use of AI in hospital medicine practice is ethically sound. Powerful AI models are challenging established ethical norms of patient data ownership, privacy, and security,^4^, ^11^ since the sheer scope of data needed for AI applications is changing how healthcare data is acquired, used, and maintained.^11^, ^12^, ^13^ As a result, hospitalists will need to understand and be able to explain the ethical implications of these changes to patients and their families in service of transparency and patient trust. Moreover, AI applications in hospital medicine will change clinical practice and how hospitalists pursue high‐quality patient‐centered care. Hospitalists will need to be active agents in monitoring whether changes in clinical practice also challenge established ethical norms of providing care that benefits patients, minimizes harm, and respects their values and preferences. Recognizing the ethical implications of AI will help hospitalists responsibly use AI to promote patients' interests, and this editorial discusses a few of the current ethical challenges of AI and the hospitalists' role in addressing them.

## HEALTHCARE DATA, INFORMED CONSENT, AND PATIENT AUTONOMY

One of the major promises of AI is its ability to identify valuable patterns in routinely generated healthcare data (i.e., prescribing, demographic, clinical, biospecimen, communication, etc.). Based on those patterns, AI can generate new data to answer medical questions and/or improve clinical tasks. For decades, the ethical use of patient data has been legitimated by the principle of informed consent, which requires patients to receive relevant information and be provided a meaningful choice about the use of their data. *Informed consent* makes it ethically permissible to use a patient's data because a patient's right and ability to decide if and how their data is used allows for self‐determination—a central tenet of the ethical principle of *patient autonomy*.^3^, ^13^, ^14^ Before AI, this ethical norm was being challenged by the social value of and impracticability of obtaining informed consent for large amounts of healthcare data. Due to its requirement for vast amounts of healthcare data, AI will almost certainly accelerate the challenge to *informed consent* and erode current norms that underpin the ethical use of patient data.^15^ At times, hospitalists may be responsible for articulating to patients who may not be fully aware of this shift in practice, how their health data is being used, and what safeguards are in place to protect it. These conversations will be necessary to maintain trust and mitigate losses in *patient autonomy*, especially given there are larger social debates occurring about trust in healthcare.

This raises another important question: As AI is increasingly integrated into and relied on to deliver patient care, will hospitalists be required or able to obtain *informed consent* for its use in directing patient care? It is already the case that patient consent *is not required* when health systems deploy clinical decision support (CDS) tools embedded within electronic health. It seems likely that AI, whether used as a CDS tool or for other purposes, will also be deployed and used without patients knowing or being able to “opt‐out” of its use in their care. This could be ethically problematic given bias can be embedded within AI products “trained” on nonrepresentative data sets that are not generalizable to all patient populations.^16^ In these instances, AI products could potentially undermine not just the ethical principle of *informed consent* but also *non‐maleficence (i.e., do no harm)* if it results in adverse patient outcomes, and/or *justice* if it inadvertently reinforces inequitable health outcomes.

## HOSPITALISTS' ACCOUNTABILITY AND RESPONSIBILITY AS MORAL AGENTS

Although AI has the potential to improve clinical decision‐making, even the most advanced AI applications will not be able to eliminate all clinical uncertainty. AI applications are built from large datasets. Most datasets have inaccuracies, are incomplete and missing data, and are not representative of all patient populations. Translating complex data with a statistical model to answer a clinical question results in a range of possible outcomes or “answers” with different probabilities.^17^ The range of possible answers, their accuracy, validity, conclusiveness, and the probability of any one answer being “optimal” in a single patient is related to the quality of the data, and the similarity of an individual patient to those in the underlying data. This will be true of even the most advanced AI applications, which use statistical models to translate complex data.^18^ Evidence‐based decision‐making requires appraising the evidence by recognizing the limitations of the underlying data, the statistical uncertainty in the possible “answer” choices, and then balancing the risks and benefits of the different clinical decisions. Hospitalists do this regularly in routine care, and this process is rooted in the ethical principles of *beneficence* and *non‐maleficence*. Although AI will be able to produce highly accurate and valid answers to clinical questions, it may not be obvious what the probability is of any answer being optimal for any given patient. Additionally, the range of possible answers and their probabilities may not be discernible when AI recommends a single answer to a clinical question. As AI becomes more ubiquitous in the clinical setting, it may be easy for hospitalists to be overconfident in and reliant on AI recommendations and not be concerned with how an AI application arrived at a specific recommendation. Yet, a blind reliance on AI to provide optimal care could result in harm (i.e., *nonmaleficence)* to patients through misdiagnosis or mistreatment. Because of this, hospitalists need to recognize AI's limitations in making clinical decisions and remain active agents integrating clinical expertise into AI outputs to optimize *beneficence* and minimize *nonmaleficence*.

AI will also be limited in that it will not be able to answer value‐laden clinical questions that are central to providing ethically sound patient‐centered care. For example, AI can inform clinicians and patients about the quantitative tradeoffs of different decisions and provide relevant acceptable ethical frameworks for making such decisions. However, which risks to accept for what benefits for a patient, is not an empirical question but rather a normative one (e.g., questions that explore what should be done to promote “good”). There is no single right “answer” to normative questions, which are often subjective and based on patients' values and preferences. For example, in two patients with identical clinical circumstances, it may be ethically permissible to forgo a life‐sustaining intervention in one patient but to provide the treatment in the other. The ethical permissibility of such a decision is based not only on medical facts but also on patient's authentic values and preferences, how they value and perceive their quality of life, and contextual features of their life that are important to them (e.g., tolerance for risk, etc.).^19^ These considerations, unique to each patient, are necessary to arrive at an ethically acceptable decision because they respect *patient autonomy*, promote *beneficence*, and minimize *non‐maleficence* for an individual patient. To answer value‐laden clinical questions such as these, hospitalists should utilize the computational strengths of AI, judiciously augment it with their clinical experience, incorporate the patient's values and wishes, and use shared decision‐making as a means to promote optimal patient outcomes.

## CONCLUSION

AI will be a part of the practice of hospital medicine. Knowing this, hospitalists should recognize that the best clinical medicine requires a balance of scientific and technical expertise in the context of the doctor–patient relationship while relying on ethical principles to pursue the trust of patients and seek their well‐being.^20^ Undoubtedly, presently unknown and unanticipated AI applications may have the greatest impact on the field of hospital medicine. Given this, clinicians will need to remain facile in their application of ethical principles. It will be easy for hospitalists to embrace the AI fervor and appreciate its benefits without stopping to consider the ethics behind its use and implementation. However, front‐line providers will have an important voice in engaging the ethical questions around AI and can make important contributions to the development of AI and its utility in medicine.

## CONFLICT OF INTEREST STATEMENT

The authors declare no conflict of interest.
